# Percutaneous triangular stabilization of type 3 and type 4 fragility fractures of the pelvis usually leads to fracture healing despite high revision rates

**DOI:** 10.3205/iprs000149

**Published:** 2020-12-16

**Authors:** Matthias Spalteholz, Jens Gulow

**Affiliations:** 1 Department of Spine Surgery, Helios Park-Klinikum Leipzig, Germany

## Abstract

This is a monocentric, retrospective study to analyze radiological findings as well as perioperative and postoperative complications in patients who underwent percutaneous triangular stabilization of type 3 and type 4 fragility fractures of the pelvis. From August 2017 to December 2018, 20 patients were treated surgically. Thirteen patients (65%) were followed-up and received a CT scan of the pelvis after an average time of 14.8 months. A total of 5 patients (38%) had to undergo revision surgery, 2 patients (15%) immediately, 3 patients (23%) in the interval. In 84.6% no fracture line was visible in the sacrum. Fracture healing of the anterior pelvic ring was observed in all cases. Our results show that percutaneous triangular stabilization of type 3 and type 4 fragility fractures of the pelvis usually leads to fracture healing. Radiological signs of loosening were observed in 62%, an implant removal due to symptomatic loosening was necessary in 23%.

## Zusammenfassung

Es handelt sich um eine monozentrische retrospektive Kohortenstudie, in der radiologische Parameter sowie perioperative und postoperative Komplikationen bei Patienten analysiert wurden, die aufgrund einer Fragilitätsfraktur des Beckens vom Typ FFP 3c und FFP 4b mittels perkutaner triangulärer Stabilisierung versorgt wurden. Insgesamt wurden von August 2017 bis Dezember 2018 20 Patienten mit FFP 3- und FFP 4-Frakturen operativ versorgt. 13 Patienten (65%) konnten nachuntersucht werden und erhielten durchschnittlich nach 14,8 Monaten eine Becken-CT. Insgesamt erfolgte bei 5 Patienten (38%) eine operative Revision, bei 2 Patienten (15%) während des initialen stationären Aufenthaltes, bei 3 Patienten (23%) im Intervall. Bei 84,6% der nachuntersuchten Patienten war im Sakrum keine Frakturlinie mehr sichtbar, eine knöcherne Konsolidierung des vorderen Beckenrings war bei allen Frakturen zu verzeichnen. Die Ergebnisse dieser Untersuchung zeigen, dass durch die perkutane trianguläre Stabilisierung eine Frakturheilung erreicht wird. Bei 62% konnten radiologische Lockerungszeichen festgestellt werden, eine Metallentfernung bei symptomatischer Lockerung erfolgte bei 3 Patienten (23%).

## Introduction

Osteoporotic fractures are in the focus of trauma and orthopedic surgeons in recent years. The confrontation of practitioners with these injuries will continue to increase due to the demographic change and an increasing level of all day activities and claim in the elderly [1], [2]. In this regard, fragility fractures of the pelvis represent a serious challenge, not only because of a significant 1-year mortality, which is up to 27% [1], [3]. Since this injury is an independent entity that cannot be compared with pelvic ring fractures in high-energy trauma patients, a corresponding classification system was inaugurated that is becoming increasingly popular – the fragility fractures of the pelvis classification (FFP classification) [2], [4]. Even if the understanding of these injuries has increased in recent years and diagnostic standards have been developed, a consistent therapy is missing. It is generally accepted that unstable fractures are treated surgically, stable fractures primarily conservatively. Various surgical procedures and strategies are listed in the literature, a uniform concept is not postulated. The authors of this article demonstrated their concept for unstable type 3 and type 4 fragility fractures of the pelvis, and established this as a treatment strategy for these types of fracture [5]. The aim of this study is to examine radiological parameters and treatment complications in the early course.

## Patients and methods

This is a single-center retrospective cohort study in which radiological parameters as well as peri- and postoperative complications were evaluated in patients who underwent triangular stabilization of type 3 and type 4 fragility fracture of the pelvis between August 2017 and December 2018. The percutaneous stabilization was performed unilaterally in type 3 fractures and bilaterally in type 4 FFP fractures [5]. All patient-related data were obtained by evaluating the electronic patient record: general patient data (e.g. age, sex, secondary diseases), perioperative and postoperative complications. The X-ray and CT image analyzation was performed by the authors and by a radiologist. The following has been analyzed: fracture healing, screw position, signs of loosening, adjacent/subsequent fractures, adjacent segment disease.

## Results

**Figure 1:**
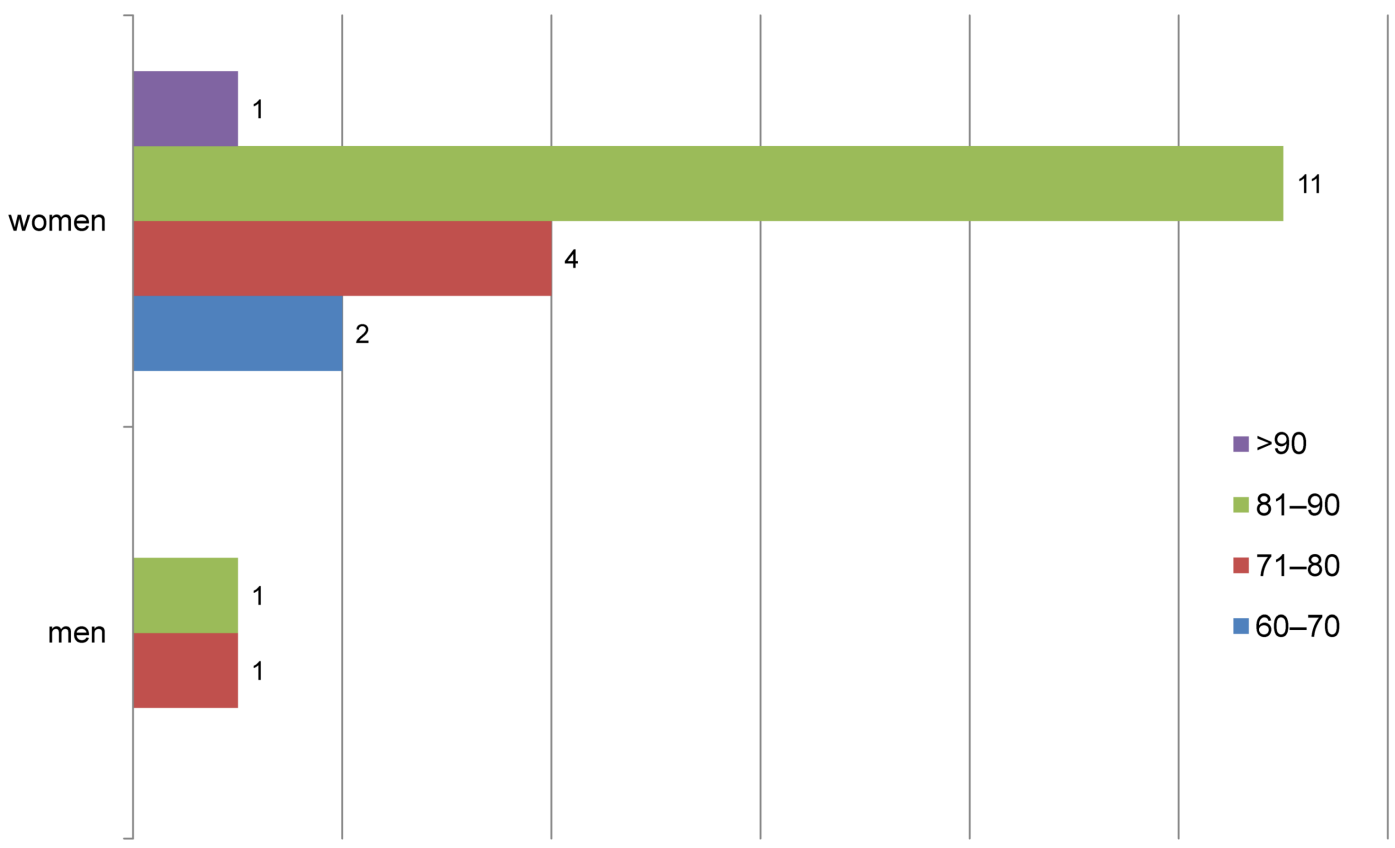
Sex and age distribution

From August 2017 to December 2018, a total of 20 patients with FFP 3 and FFP 4 fractures were treated surgically (18 women, 2 men). The mean age was 82.3 years (χmed: 84) (Figure 1 (Fig. 1)). 13 patients (65%) could be included in this study. Pelvic CT examination was performed after an average of 14.8 months (χmed: 15). Until January 2019 (starting point of data analysis), 4 patients (20%) died (one patient died 9 months after surgery due to an advanced tumor disease; she received an abdominal CT scan 6 months after surgery due to abdominal problems, which was analyzed for this study). 4 patients were not reached (20%).

### General patient data

#### Secondary diagnoses

**Figure 2:**
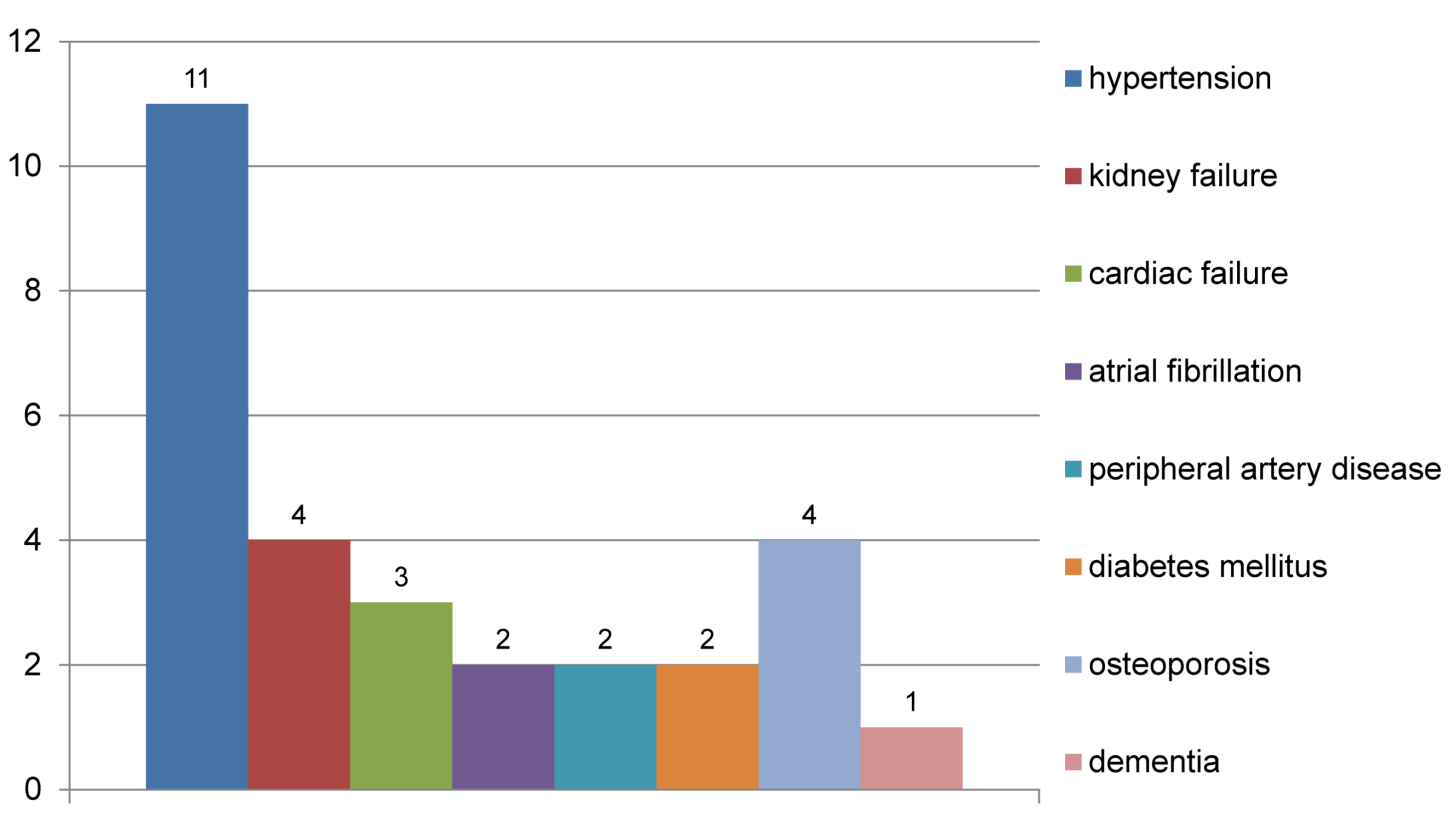
Secondary diseases

Every patient had at least one documented secondary disease that required treatment. Cardiovascular diseases were the most common. Figure 2 (Fig. 2) shows the secondary diagnoses.

#### Osteoporosis therapy at admission

Osteoporosis was documented in 4 patients. 2 patients received a specific bisphosphonate therapy. 7 patients received a calcium/vit. D3 supplementary therapy. 11 patients did not receive medication to support bone metabolism.

#### Malignant diseases

5 patients had a history of malignant disease: bladder carcinoma (palliative), renal cell carcinoma (curative, tumor nephrectomy), breast carcinoma (palliative), rectal carcinoma (palliative), multiple myeloma.

#### Cause of fracture

14 patients reported a fall (70%). 12 of these patients reported a trivial fall on the buttocks or hips (60%). 2 patients reported a low energy bicycle accident (19%). 6 patients reported no event (30%).

#### Period of time until admission

10 patients (50%) were presented immediately by paramedics with immobilizing pain after the fall. 10 patients presented with delay via the emergency room or the outpatient clinic, on average after 7 weeks, due to persistent pain (χmed: 8; min 2 weeks; max 12 weeks). All atraumatic fractures (six) presented with a delay.

#### Fracture type

**Figure 3:**
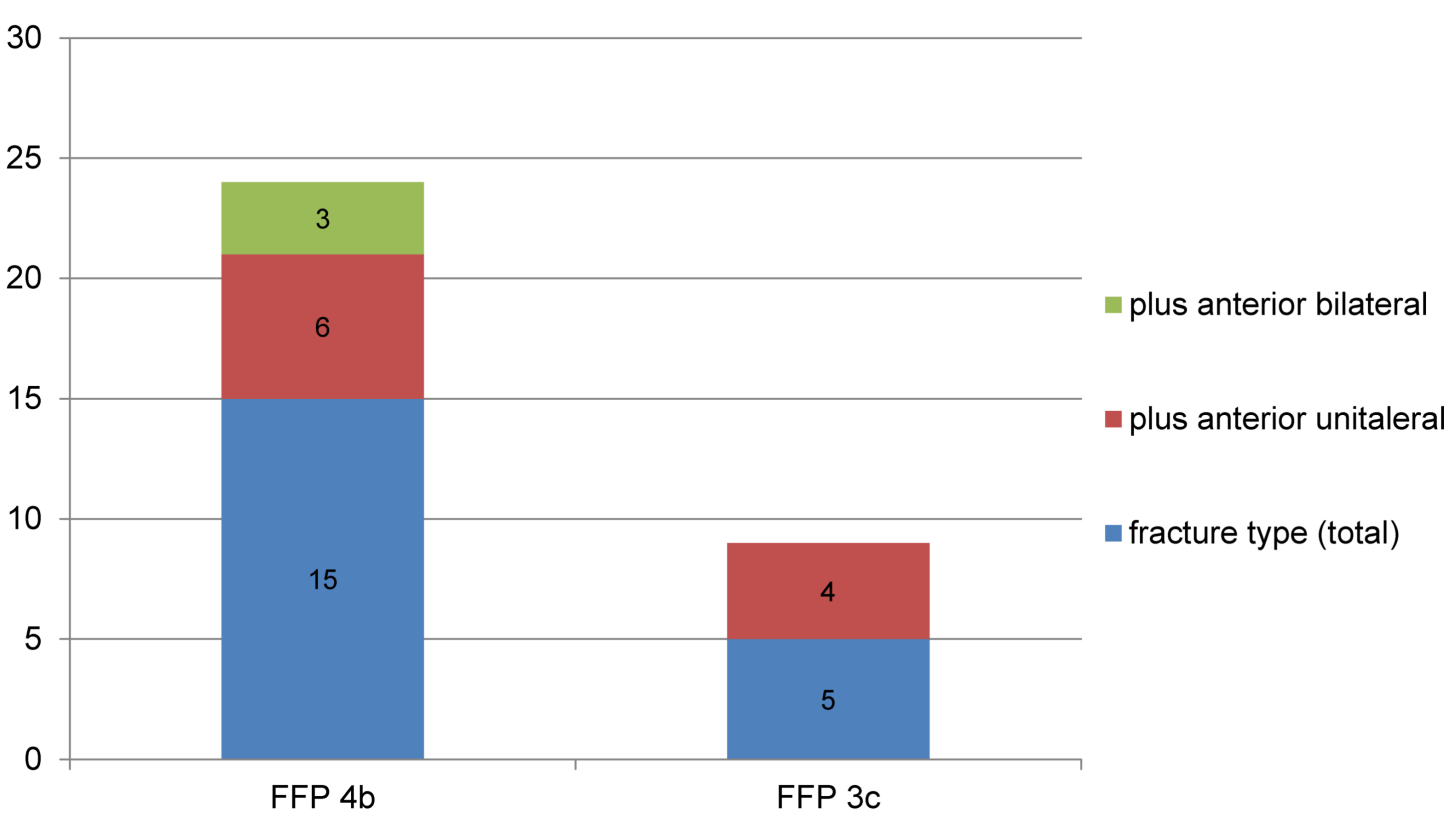
Fracture type

15 patients had a type 4b fracture and 5 patients a type 3c fracture, regarding the fragility fracture of the pelvis classification. In type FFP 4 fractures, the anterior pelvic ring was involved in 60%, bilaterally in 20%. 87% of the type FFP 4b fractures were H-type-fractures with the horizontal fracture component running through the second sacral body. In FFP 3c type fractures, the anterior pelvic ring was involved in 80%, only unilaterally, ipsilaterally (Figure 3 (Fig. 3)).

#### Period of time from admission until surgery

The average preoperative length of stay was 5.4 days (χmed: 5). Eleven patients were admitted directly into the spine department (clinic of the authors) via the emergency room. In this case, the preoperative length of stay was 3.2 days in average. Seven patients were admitted into the geriatric department for a geriatric complex therapy/rehab. In this case, the average preoperative length of stay was 8.9 days. Two days after admission into the spine department and surgery, two patients were shifted to the geriatric department for a complex therapy.

#### Duration of hospital stay

The average length of stay was 15.1 days for all patients (χmed: 15.5). Patients who were treated in the spine department exclusively had an average length of stay of 11.5 days. Patients treated in the geriatric department had an average length of stay of 19.5 days.

#### Discharge management

Of the 11 patients who were admitted into the spine department, 10 patients were directly discharged into the geriatric rehabilitation. One patient was discharged into nursing home due to missing capability for rehabilitation. The 9 patients who were treated in the geriatric department were discharged after completing the complex geriatric therapy (home: 7, nursing home: 2).

#### Revision surgery

5 of the 13 followed-up patients (38%) needed revision surgery. In two cases (15%) revision surgery was performed during the initial hospital stay, in 3 cases (23%) in the interval (after 4 months, after 12 months, after 23 months).

#### Complications and revision surgery during the hospital stay

**Figure 4:**
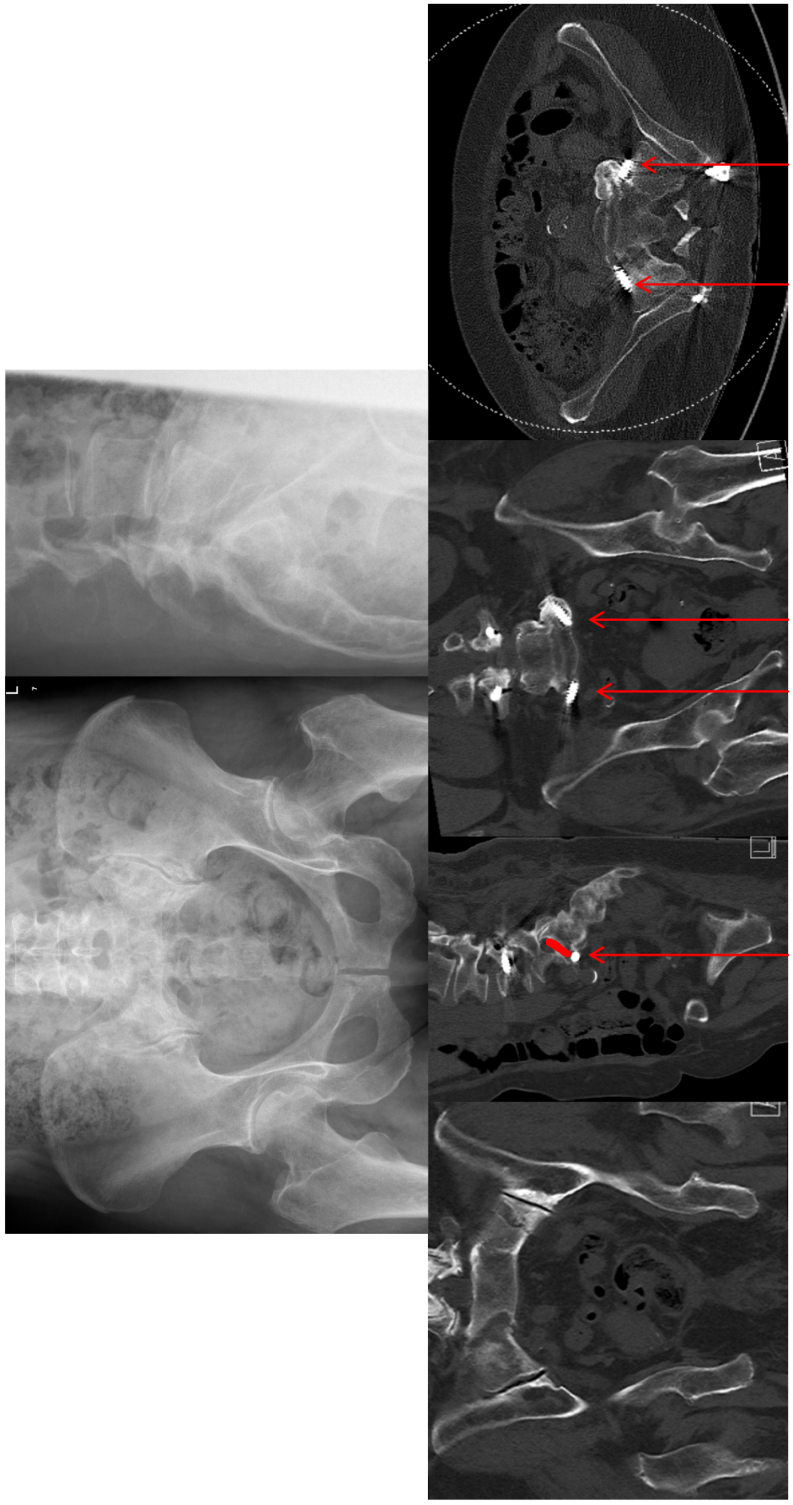
FFP 4b type fracture. The red arrows show the malposition of the sacroiliac screws anterior to the ala. The short red line shows the course of the L5 nerve. The conflict between the screws and the nerves is obviously.

Revision surgery was necessary in two patients during the hospital stay. One patient with a FFP 4b type fracture presented a drop foot, grade 4 according to BMRC (British Medical Research Council), due to a malposition of the sacroiliac screws. Due to progressive paralysis (grade 3) and progressive Trendelenburg sign, revision surgery was performed five days after primary surgery. 1 year after revision surgery the paralysis was declining (grade 4), there was no pain (Figure 4 (Fig. 4)). 

One patient with a FFP 3c type fracture on the left side and unilateral triangular stabilization demonstrated a pedicle screw pull out at L4 vertebral body with progressive pain. Revision surgery with re-instrumentation was necessary 7 days after primary surgery.

#### Revision surgery in the interval

3 out of the 13 followed-up patients (23%) required an implant removal due to painful implant loosening. One patient complained of dysesthesia in the pelvic area, no pain. The CT control after 23 months showed a loosening of the pedicle and iliac screws. The implant was removed completely. One patient had to undergo revision surgery 4 months after primary surgery due to a pedicle screw pull out in the L4 vertebral body. The CT scan had not yet shown any fracture healing in the sacrum. After revision surgery with extension of the instrumentation up to the L3 vertebral body and renewed screw dislocation, the internal fixator was removed 6 months after the revision surgery. The CT scan showed secure fracture healing at this time. The iliosacral screws were not removed. One patient complained of pain due to loosened and displaced sacroiliac screws. The internal fixator showed no signs of loosening. A complete implant removal was performed one year after surgery. 

### Radiological assessment

#### CT evaluation – implant loosening

**Figure 5:**
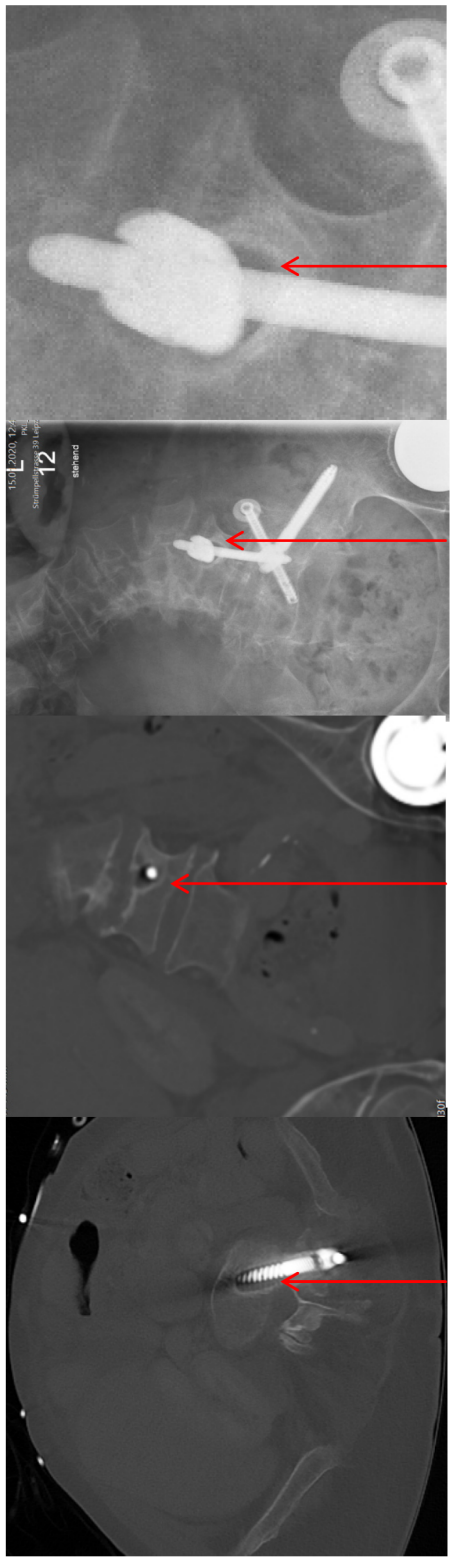
FFP 3c type fracture and unilateral triangular stabilization. The red arrows mark the radiolucent area around the pedicle screw in the L4 vertebral body.

**Figure 6:**
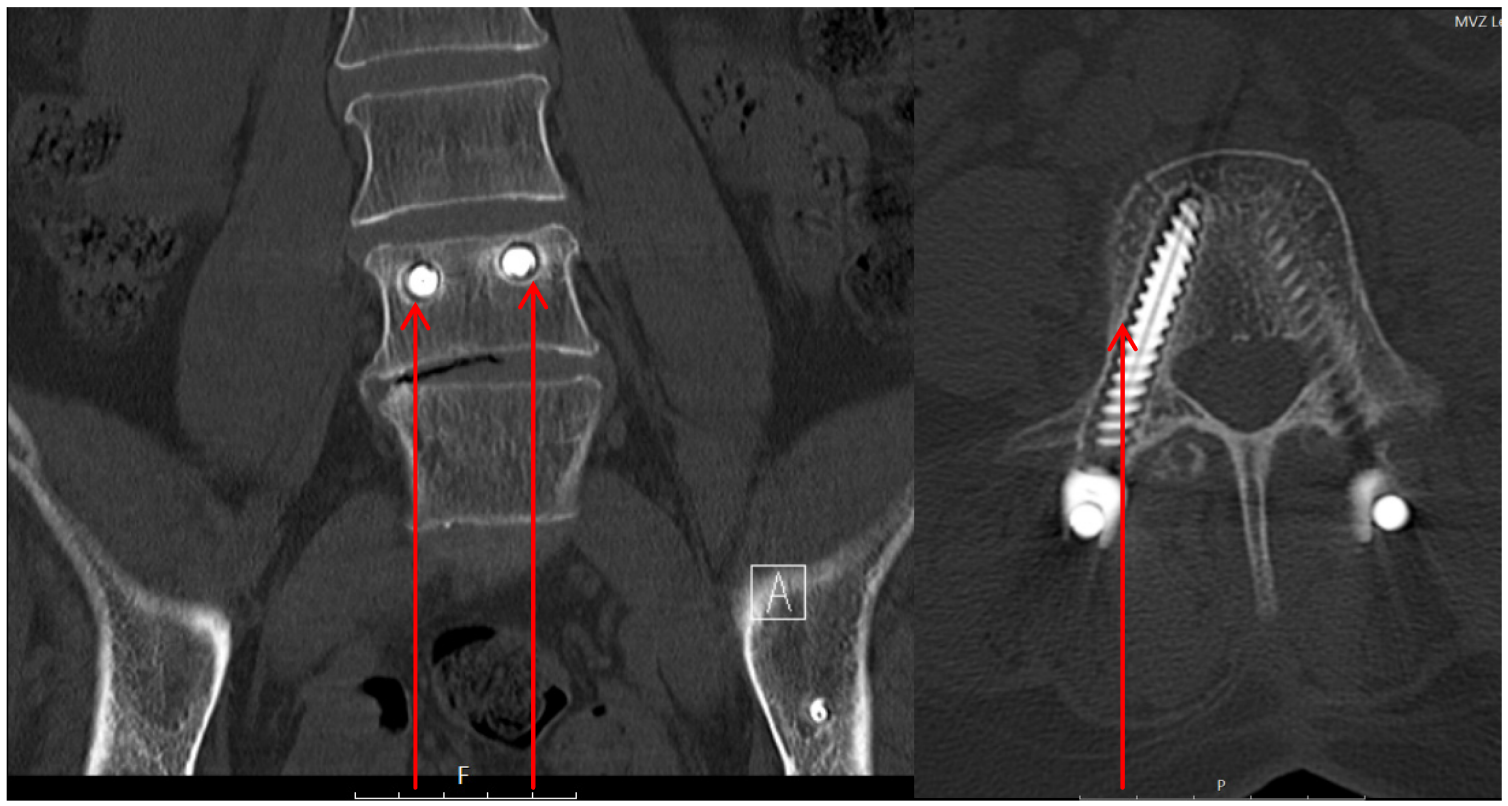
The red arrows mark the “double halo” sign around the L4 pedicle screw. One can find clearly the radiolucent area and radiopaque rim.

CT criteria for implant loosening included a radiolucent area (thicker than 1 mm) around the screw (Figure 5 (Fig. 5)) and the “double halo” sign (Figure 6 (Fig. 6)). The “double halo” sign is defined as the presence of a radiolucent area and a radiopaque rim at the same image [6]. Radiological signs of loosening were found in 8 of 13 CTs (62%). A radiolucent area >1 mm was present in all 8 patients (pedicle screws and iliac screws). 7 CTs (54%) showed a “double halo” sign (only iliac screw: 1 patient, only pedicle screws: 2 patients, pedicle and iliac screws: 3 patients, pedicle, iliac and iliosacral screws: 1 patient). A screw dislocation was seen in 4 patients (pedicle screws: 2 patients; iliosacral screws: 2 patients).

#### CT evaluation – fracture healing

**Figure 7:**
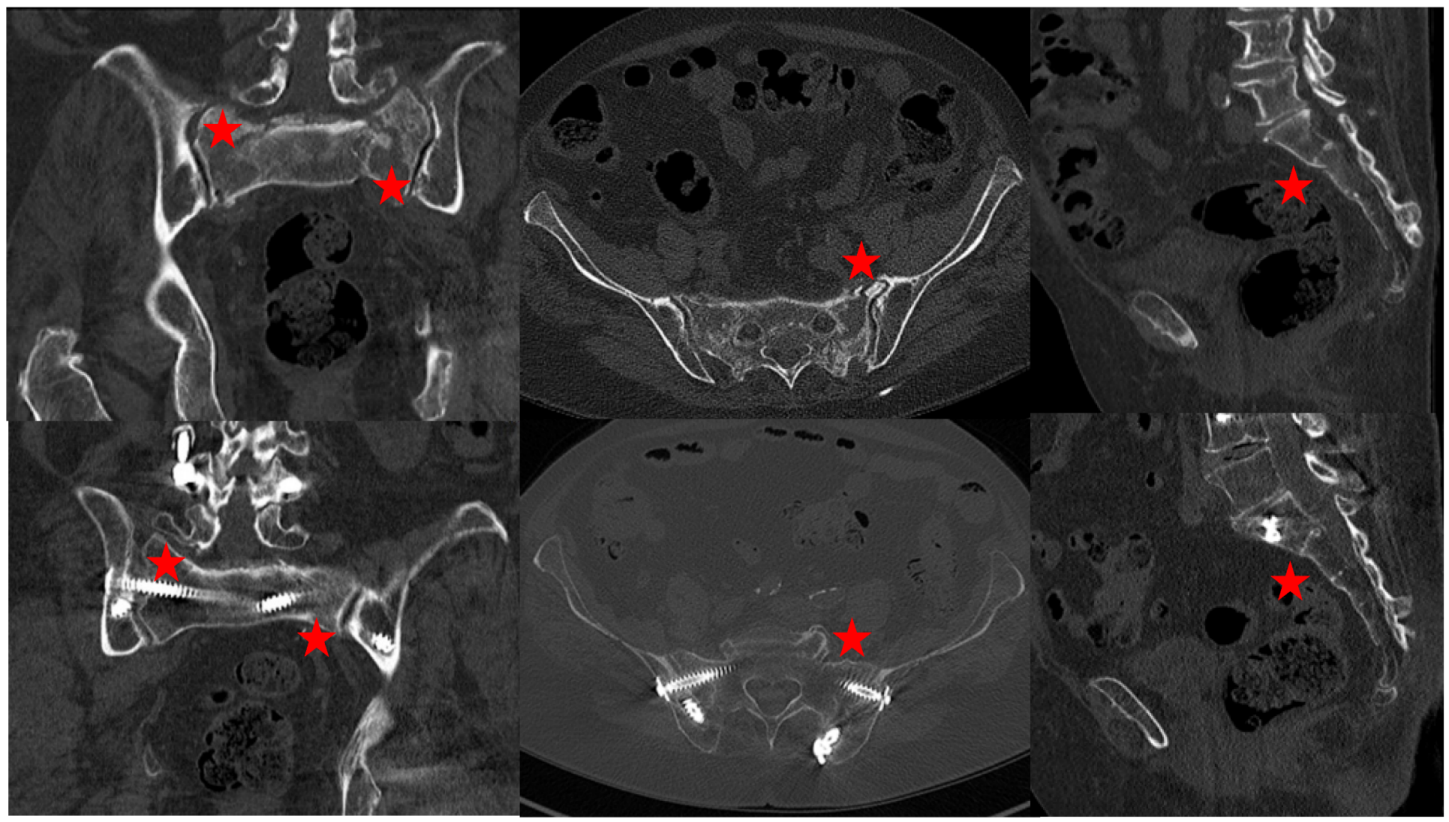
Top row: FFP4b type fracture. The stars mark the fracture zones. Bottom row: bony fusion after triangular stabilization. The stars mark the former fracture zone. No fracture lines visible.

Criteria for bony fusion were visible fracture lines in the anterior and posterior pelvic ring, sclerosis and callus formation (Figure 7 (Fig. 7)). In 11 of the 13 followed-up patients, no fracture line was visible in the sacrum (84.6%). An H-type fracture was present in 11 patients, a visible callus formation at the second sacral vertebral body (horizontal fracture line) was seen in 7 patients. A callus formation was seen in every case of an anterior pelvic ring fracture. Subsequent and adjacent fractures were not seen in any case. Adjacent segment degeneration was found in one patient (at the level L3/4, asymptomatic). 

## Discussion

The FFP classification postulates stable and unstable fractures based on radiological findings, which should result in a treatment recommendation. FFP 3 and FFP 4 type fractures are considered to be unstable and should be treated surgically [4], [7]. Since 30% of geriatric pelvic ring fractures are classified as FFP 3 and FFP 4 type fractures, the need for surgery in these patients is correspondingly high [4]. There is no consensus on the type and extent of surgery. The aim of this work is to assess fracture healing and possible implant complications after percutaneous triangular stabilization of FFP 3 and FFP 4 fractures. It could be demonstrated that fracture healing occurred in 84.6% of the patients after an average time of 14.8 months (mean time of CT FU). Radiological signs of implant loosening were observed in 62%. Consistent implant removal was necessary in 3 patients (23%). 

The treatment concept for fragility fractures of the pelvis continues to be controversial [8], [9], [10]. Most authors recommend surgical treatment for unstable fractures in order to reduce pain and enable early mobilization [11], [12], [13], [14]. Currently, there is no evidence that surgery is superior to conservative treatment. In addition, there is no evidence regarding the best operative strategy, because the term instability has not yet been adequately defined in geriatric pelvic ring fractures. The FFP classification is intended to be a decision-making aid [4], but which type of surgery should be used is not explicitly postulated. The rationale for surgery must be the sufficient stabilization of the pelvic ring in order to avoid persistent instabilities, deformities and secondary nerve lesions. In the opinion of the authors, the main reason for the triangular stabilization (lumbopelvic and iliosacral fixation) of the posterior pelvic ring is its superior biomechanical results compared to other procedures [15]. Early mobilization under full load is guaranteed. The pitfalls of this procedure have been described [5]. Particularly it is important to ensure that the iliosacral screw is in the center of the sacrum or reaches the promontory, as this is where the bone quality is best [16]. To avoid perforation of the lateral ilium by the screw head, washers should be used. In case of osteoporotic bone conditions, cement augmentation is recommended to minimize the risk of screw loosening and screw dislocation [12], [17]. Various aspects must be considered when screwing the iliac bone [5]. The direction of the iliac screw (direction onto the AIIS or supraacetabular) is not decisive for the fixation strength of the iliac screw, but the size of the implant. Screws with a diameter >9.5 mm and a length >80 mm showed a significantly more stable fixation [18]. The advantages of a percutaneous screw implantation are faced by the risk of vascular and nerve lesions as well as malpositions. In some cases, the radiological landmarks cannot be identified exactly due to overlapping effects in conventional X-rays [19]. Therefore some authors recommend intraoperative 3D imaging or navigation [5], [20]. In our collective, one patient demonstrated a drop foot after surgery, due to malposition of an iliosacral screw. In this case, both screws were located in front of the sacral ala and resulted in an unilateral L5 nerve root compression. Revision surgery and screw correction (new instrumentation both sides) was necessary. This was one of the main reasons why we optimized our surgical technique. Since then, we perform the percutaneous stabilization of the pelvic ring by using intraoperative 3D imaging, exclusively: after placing the guide wire, the 3D scan is performed and the screw implantation is done afterwards. Using this technique, screw malposition and neurological complication no longer occurred [5]. The high number of implant loosening must be taken into account, the clinical relevance is currently not clarified. Ohtori et al. observed radiological signs of implant loosening 12 months after lumbar fusion surgery in osteoporotic bone in 7–15% in X-ray examination and 13–26% in CT scans [21]. Other studies even describe loosening rates in osteoporotic bone conditions in up to 60% of cases [22]. In many cases, however, this is only a radiological finding. Wu et al. did not find a significant difference in VAS back pain, VAS leg pain or ODI scores compared to the control group without screw loosening after 24 months in any patient with obviously pedicle screw loosening [23]. Röllinghoff et al. demonstrated implant loosening in 54% after spinal fusion surgery, with 20% complaining of back pain, compared to 11% of the entire cohort [24]. In our collective, implant loosening was found in 62% leading to consequences in 23% (implant removal). 

Besides the triangular instrumentation, further procedures are described for the surgical treatment of geriatric pelvic ring fractures. The results of the conventional surgery of the posterior pelvic ring – so called open reduction and internal fixation – show significantly higher morbidity than minimally invasive or percutaneous procedures. These are more complex interventions with longer operating times and increased blood loss [15]. The infection rate is up to 16% [25]. Painfully implant prominence is reported in up to 95% [26], which also leads to soft tissue irritation [27]. Percutaneous iliosacral screwing is the standard procedure for stabilizing the posterior pelvic ring, especially in case of insufficiency fractures of the sacrum. Studies have shown an adequate reduction in pain and an improvement in mobilization [9]. The sacroiliac screw fixation can be performed as compression screw osteosynthesis (partial thread) or as adjusting screw osteosynthesis (full thread). This is mandatory in case of fractures involving the neuroforamen, to avoid foraminal compression and nerve root lesion. The screws can be placed into the S1 and S2 body, with 2 screws providing major stability. Screw augmentation increases fixation strength in osteoporotic bone [28]. Considering anatomical conditions is crucial for secure screw implantation, attention must be paid to variations of the sacrum morphology [29]. The complication rates are low. Hopf et al. recorded one screw malposition with nerve root irritation, two gluteal hematomas requiring revision and one screw loosening with required implant removal in a collective of 30 patients [17]. Similar results are reported in other studies [9], [11], [30]. However, it should be mentioned that these results are partly based on publications relating to the treatment of traumatic pelvic ring fractures in younger patients [9], [11], [30]. In addition, some studies talk about fracture instability without precisely defining it (no specification according to the FFP classification) [17]. In the current literature it is not proven that sacroiliac screw fixation is a safe procedure for FFP 3 and FFP 4 fracture stabilization. There is a lack of reliable data to demonstrate the evidence of sacroiliac screw stabilization in these kinds of fractures. 

Further surgical procedures are described to stabilize fragility fractures of the pelvis, which are briefly listed below. The current data do not allow any statement about the superiority of one procedure. The sacroplasty is a percutaneous procedure, in which a cavity is created in the ala ossis sacri, which is augmented with cement, like in kyphoplasty. This leads to intraosseous stabilization and proven pain reduction. The risk of cement extrusion must be taken into account, in which the information in the literature varies between 0.4% and 27%. Because fracture healing is prevented, this technique should not be used in unstable fractures [31], [32]. The posterior iliac plate fixation (transiliac bridging osteosynthesis) can cause soft tissue problems (internal decubitus ulcer) and therefore must be bent precisely to the contour of the posterior pelvic ring. Angle-stable plates can be used in osteoporotic bones (internal fixator) in a minimal-invasive fashion (“MIPPO technique”) [27]. The iIiac screw fixator is another possibility for posterior pelvic ring stabilization. Polyaxial screws are inserted into the posterior superior iliac spine and connected to a subfascial bar [33]. The transsacral bar osteosynthesis is another minimally invasive procedure. A threaded rod is inserted from the ipsilateral to the contralateral posterior ilium through the transsacral corridor of S1 [34]. The combination with a iliosacral screw is possible [35]. The stability of the construct depends on the stability of the outer cortex of the posterior ilium. Penetration of the threaded screws into/through the iliac bone must be avoided. CT analysis to evaluate anatomical variations is indispensable [35].

Irrespective of the surgical strategy, it should be noted that 60% of the fragility fractures of the pelvis are osteoporosis related fractures [3], [2], [36], [37]. Therefore a specific osteoporosis therapy is mandatory. Antiresorptive substances such as bisphosphonates are well established. New therapeutic approaches with the recombinant parathyroid hormone 1-84 fragment analogue teriparatide (Forsteo^®^) have demonstrated a significantly reduced healing time for osteoporotic pelvic fractures [38], [39].

## Conclusion

Our results show that the percutaneous triangular stabilization of type 3 and type 4 fragility fractures of the pelvis usually leads to fracture healing. Implant loosening occurs, but revision surgery is required in a few cases only, usually implant removal. In summary, the authors state that, on the basis of the results of this work and the current evidence, an unitarian treatment strategy, depending solely on the fracture type, should be critically assessed. The current results of this analysis prompt the authors to think about their treatment concept and even more intensively to choose a patient-specific approach. From the point of view of the authors, further studies are necessary, in particular with the inclusion of patient-specific parameters, in order to establish a reliable treatment algorithm for pelvic fragility fractures.

## Limitations

The major limitation is the low number of cases. This is due to the fact that it is a selected patient group. About 70% of the fragility fractures of the pelvis (FFP I and FFP II type fractures) are primarily treated conservatively. In case of FFP type II fractures that need surgery, we do not perform the above mentioned technique, but an iliosacral screw fixation. Another weakness is the poor follow-up, which is partly due to the high 1-year mortality in this entity. Further, residence change after leaving the hospital (nursing home, relatives) is a major factor in the lack of reachability. The absence of subjective parameters (PROMs, COMIs) is due to the missing participation in the outpatient consultations. Only 7 patients (35%) joined the recommended 3-month control. Therefore follow-up documentations were not available.

## Abbreviations

- AIIS = anterior inferior iliac spine
- BMRC = British Medical Research Council
- COMI = core outcome measure index
- FFP = fragility fractures of the pelvis
- max = maximal
- min = minimal
- ODI = Oswestry Disability Index
- PROM = patient reported outcome measures
- CT = computed tomography
- VAS = visual analogue scale

## Notes

### Competing interests

The authors declare that they have no competing interests.
